# How Can We Improve the Care of Severely Malnourished Children in Africa?

**DOI:** 10.1371/journal.pmed.0040045

**Published:** 2007-01-30

**Authors:** Geert Tom Heikens

## Abstract

Heikens discusses a new study published in*PLoS Medicine* that is helpful in reconsidering the applicability of the WHO treatment guidelines.

More than 10 million children under the age of five die each year, of whom 1.5 million are severely malnourished [1]. Pelletier and colleagues showed that 53%–60% of global child deaths are attributed to malnutrition (determined by weight-for-height z-scores of less than minus 1) [2], representing 5.7–6.4 million malnutrition-related deaths each year associated with pneumonia, diarrhoea, measles, and malaria [3].

Community-based programs, including the Integrated Management of Childhood Illness initiative of the World Health Organization (WHO) (see http://www.who.int/child-adolescent-health/integr.htm), have been developed to address the synergy between childhood malnutrition and infection, and these programs include nutritional rehabilitation [4]. Such programs detect severely malnourished children by measuring mid-upper arm circumference, treat such children with ready-to-use therapeutic foods [5], and if necessary refer them to facility-based management. This is a cost-effective approach for secondary prevention and treatment of severe malnutrition [6,7]. A small proportion of severely malnourished children (15%) are very sick and require hospitalisation.

## WHO's Treatment Guidelines for Severe Malnutrition in Children

The WHO therapeutic guidelines [8,9] are based on a large body of accumulated experience of over 30 years, from nutrition units in Uganda [10], South Africa [11], and the Caribbean [12,13]. And yet despite this extensive clinical experience and physiological reasoning, there have been very few clinical trials.

Since 1970 there has been vigorous debate over where and how to optimally rehabilitate malnourished children [14–16]. The mortality risk of these children is thought to relate to several factors [17], including electrolyte imbalance [18], hepatic dysfunction, infection, anthropometric status [19], and micronutrient status, as well as to differences between treatment regimens. The pathophysiology of *primary* malnutrition [20] and kwashiorkor [21] is to a large extent understood and has informed treatment guidelines improving the case fatality from primary malnutrition to levels below 5% [22,23]. Equally important, understanding the pathophysiology of primary malnutrition and kwashiorkor has enabled nutrition rehabilitation to grow to an unprecedented scale through humanitarian assistance and community-based programs [1,24]. Residual high mortality has been ascribed to faulty practices [25–27], but to date no published randomised controlled trials have been carried out to support these statements.

## Severe Malnutrition Due To HIV and Tuberculosis in Sub-Saharan Africa

In sub-Saharan African countries with the highest case fatality of malnutrition, AIDS and tuberculosis (TB) have led to an epidemic of *secondary* severe malnutrition related to these co-morbidities [28]. Severely sick malnourished children with AIDS and TB appear to differ in their pathophysiological and clinical response to the accepted WHO therapeutic guidelines, compared with children with primary severe malnutrition due to food shortage and non-HIV/TB related infection [29].

A WHO collaborative study [30] and other studies [31,32] assessed the successful application of WHO guidelines in sub-Saharan Africa. These studies concluded that achieving mortality rates as low as 5% was not straightforward, given the severe co-morbidity in the sub-continent, especially in the context of overloaded, demotivated, eroded, and under-resourced child health services [33–35].

## A New Study

The question arises: is it the *therapeutic approaches* that are at fault, or is it that the *population being treated* differs from those on which the WHO treatment guidelines were based? A new study published in *PLoS Medicine*, by Kathryn Maitland and colleagues, helps to answer this crucial question [36]. The study is timely and relevant for reconsidering the applicability of the WHO treatment guidelines.

The study was completed in response to the view repeatedly expressed that case fatality rates above 5% were unacceptable and could be attributed to inadequately trained health staff, poor compliance with WHO treatment guidelines, or even faulty practices. Maitland et al. conclude that there is insufficient evidence to indicate that the practices are faulty in the Kilifi setting in Kenya, yet case fatality remains considerably above 5%.

The researchers conducted a retrospective study of 920 severely malnourished children admitted to Kilifi District Hospital for clinical and nutritional rehabilitation. The retrospective design limits its generalisability. Nonetheless, the quality of care delivered by this “district hospital” [37] could be considered excellent in terms of its paediatric staff (trained in paediatric emergency triage assessment and treatment [38]), scientific experience, equipment, and laboratory services. And so the 4-fold higher case fatality rate recorded in Kilifi is not likely to result from inadequate standards of care or faulty practices.

Maitland et al. argue that better clinical characterisation with appropriate clinical treatment is required for those most at risk of complications during initial case management. In order to identify those most at risk, they studied patients' clinical and laboratory indicators upon admission and tried to relate these to possible therapeutic requirements and outcome. When WHO guidelines were followed, case fatality rates fell from 30% to 19% within a short period, demonstrating the immediate relevance of this critical care pathway. The authors studied the other factors related to the persistently raised case fatality. The prevalence of bacteraemia in the 920 children was 17%, which is similar to that in severely malnourished children studied in the Kingston Project in Jamaica [39]. A large proportion of fatalities (36%) had invasive bacterial disease.

**Box 1. Three Groups Identified by Maitland and Colleagues that Differed in Prognosis and the Need for Emergency Care**

- **Very high risk group:** This group included children with any one of the prognostic variables or hypoglycaemia. Case fatality in this group was 94/277 (34%), compared to 54/423 (12%) for children without any of these features χ^2^ = 45.0; p < 0.0001).
- **Moderate risk group:** Children without high risk features were further resolved into a moderate-risk group, with any one of the features of deep acidotic breathing, acute dehydration, lethargy, hyponatraemia, or hypokalaemia. The attendant fatality rate was 32/106 (23%).
- **Low risk group:** This group had none of the above features, and only 22/285 (7%) died.

The very poor clinical state of children who died within 48 hours of admission was not characterised by more specific diagnoses, i.e., frank sepsis, degree of oedema or marasmic kwashiorkor, or in relation to severe anaemia. Some children who died within an hour or so of admission were likely to be impossible to save. So questioning the whole basis of the current approach to stabilisation on the basis of the clinical state of such children may well be unjustified even though they may have significant co-morbidity.

In the resuscitation phase, the factors associated with and strongly predictive of a fatal outcome were: (1) bradycardia, (2) reduced conscious level, (3) capillary refill time >2 seconds (according to the Advanced Pediatric Life Support guidelines, rather than >3 seconds as recommended by WHO [40]), (4) a weak pulse, and (5) hypoglycaemia (a WHO manual criterion for immediate intervention). Other clinical risk factors were acidotic breathing, signs of dehydration, hyponatraemia, hypokalaemia, and lethargy.

Exact data on use of intravenous fluids or transfusion is scant and it is difficult to assess the use or benefit of these in relation to the clinical state of the child. The Kilifi group may have underestimated the extent of sodium excess in some of these oedematous children and the degree of impairment of the sodium pump, which is a major contributor to pathophysiology in *primary* severe malnutrition [41].

## Triaging Patients into Three Groups

Maitland et al. identify three risk groups for triage: a very high risk group, a moderate risk group, and a low risk group (Box 1), which corresponded to fatality rates of 34%, 23%, and 7%, respectively. The low risk group had no specific acute clinical signs or symptoms and needed only “limited requirements for close supervision”.

These fatality differences could indicate intrinsic pathophysiological differences between these groups of children. It is very difficult to know whether more aggressive initial management, as implied by Maitland et al., might benefit some children but equally harm others. A better understanding of the electrolyte disturbances both extra- and intra-cellular will be important in resolving these questions.

Another critical question to consider [M. J. Manary, personal communication, 2006] concerns possible missing pathophysiological elements in the concept of rehabilitation, as expressed in the WHO manual and the study by Maitland et al., which do not adequately address the sick child with multiple co-morbidity in sub-Saharan Africa and which we are still not able to comprehend. Adequate nursing [42] is critical but the paradigm of primary severe malnutrition as experienced prior to the epidemic of HIV-related disease requires reassessment with further clinical and pathophysiological studies.

## Implications for Action

I would like to suggest that five aggregated determinants characterise the unresolved problem in the case management of sick severely malnourished children in sub-Saharan Africa.

### Reductive adaptation to reduced dietary intake

Practitioners rehabilitating severely malnourished children should be aware of the important concept of reductive adaptation [43,44] which characterises severely malnourished children. Reductive adaptation involves reduced homeostasis to allow the body to economise in energy expenditure. The pathophysiological changes which occur are multiple and reserve metabolic function may be compromised, especially in infection (Table 1). As a result many aspects of metabolic control are brittle, with only a limited ability to withstand any perturbation, as may be induced during resuscitation using Advanced Paediatric Life Support guidelines.

**Table 1:**
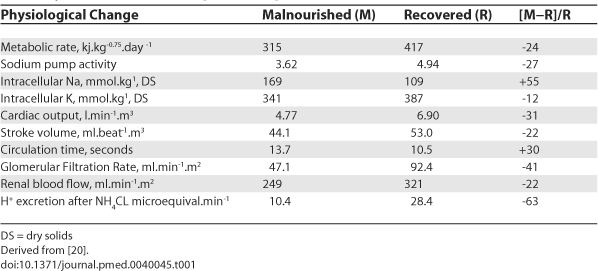
Physiological Changes in Severely Malnourished Children and Children after Recovery to Their Normal Weight for Height

### Critical care paediatrics and severe malnutrition

As severely malnourished children often present with multi-organ failure, more intensive critical care might prove beneficial but would require more complex assessments (metabolic studies, imaging, organ function) in order to establish the appropriateness of proposed new interventions.

### Assessing multiple co-morbidity

Detailed prospective clinical studies of co-morbidity are required with accurate documentation of both metabolic and clinical status, and response to different therapies, in the first few days following admission. These studies will form an essential basis for proposing changes in the early critical care of these children or in designing appropriate randomised controlled trials.

### The delivery of child health services in sub-Saharan Africa

Since the first draft of the WHO 1999 manual was circulated in the early 1990s, many specialists [45] have critically examined requirements for and delivery of good quality care in district child health services. Rather than ascribing case fatalities to faulty practices, these studies have tried to understand and describe the adverse circumstances under which such children are managed, and to address the health services' potential for improvements [46,47]. In Malawi, for instance, evidence-based triage and critical care pathways were developed and are now practised with training nationally [46,47]. This has reduced case fatality in the largest paediatric department in the country (> 26,000 annual admissions) from 10%–18% to 6%–8% [48]. For these reasons English et al. [37] and Molyneux and Webber [49] strongly argue for overall strengthening of district hospitals rather than focusing on vertical programs.

### Global understanding that severe acute malnutrition is treatable and must be made a high priority if the Millennium Development Goals are to be achieved

As argued by Collins [1] and Briend et al. [50], the global importance of severe acute malnutrition as a major cause of avoidable death needs to be better communicated as a high priority on the child survival agenda [51].

## The Way Forward

Duke et al. recently suggested that the process of WHO consultations be expanded through testing and piloting of the proposed guidelines in a variety of settings [52]. Multi-centre studies are crucial, and they must incorporate new approaches to critical care pathways in the early phase of treatment for severe acute malnutrition. This work should include appraisal of emerging bacterial resistance and its impact on mortality, and it must consider critical care pathways for resource-poor district hospitals. Only then will we be able to deliver adequate care to millions of young children whose problems have hitherto not been addressed by the child survival agenda [53].

## Abbreviations

TB: tuberculosis
WHO: World Health Organization

## References

[pmed-0040045-b001] Collins S, Dent N, Binns P, Bahwere P, Sadler K (2006). Management of severe acute malnutrition in children. Lancet.

[pmed-0040045-b002] Pelletier DI, Frongillo EA, Schroeder DG, Habicht JP (1994). A methodology for estimating the contribution of malnutrition to child mortality in developing countries. J Nutr.

[pmed-0040045-b003] Caulfield LE, de Onis M, Blossner M, Black RE (2004). Undernutrition as an underlying cause of child deaths associated with diarrhea, pneumonia, malaria and measles. Am J Clin Nutr.

[pmed-0040045-b004] World Health Organization (1999). Management of the child with a serious infection or malnutrition: Guidelines for care at the first-referral level in developing countries.

[pmed-0040045-b005] Briend A, Lascala R, Prudhon C, Mounier B, Grellety Y (1999). Ready-to-use therapeutic food for treatment of marasmus. Lancet.

[pmed-0040045-b006] Manary MJ, Ndekha MJ, Ashorn P, Maleta K, Briend A (2004). Home based therapy for severe malnutrition with ready-to-use food. Arch Dis Child.

[pmed-0040045-b007] World Health Organization, UNICEF, Standing Committee on Nutrition (2005). WHO, UNICEF and SCN informal consultation on community-based management on severe malnutrition in children.

[pmed-0040045-b008] World Health Organization (1999). Management of severe malnutrition: A manual for physicians and other senior health workers.

[pmed-0040045-b009] World Health Organization (2005). Severe malnutrition: Report of a consultation to review current literature.

[pmed-0040045-b010] Hay RW, Whitehead RG (1973). The therapy of the severely malnourished child: A practical manual.

[pmed-0040045-b011] Pretorius PJ, Hansen JD, Davel JGA, Brock JF (1956). Skimmed milk and kwashiorkor. S Afr Med J.

[pmed-0040045-b012] Picou D, Alleyne GAO, Brooke O, Kerr DS, Miller C (1978). Malnutrition and gastroenteritis in children: A manual for hospital treatment and management.

[pmed-0040045-b013] Garrow JS, Picou D, Waterlow JC (1962). The treatment and prognosis of infantile malnutrition in Jamaican children. West Ind Med J.

[pmed-0040045-b014] Jelliffe DJ (1970). The children's ward as lethal factor. J Pediatr.

[pmed-0040045-b015] Cook R (1971). Is hospital the place for the treatment of malnourished children?. J Trop Pediatr.

[pmed-0040045-b016] Heikens GT (2003). Rehabilitation of sick malnourished children: Environment, requirements, prognosis and feasibility [PhD thesis].

[pmed-0040045-b017] Garrow JS, Pike MC (1967). The short-term prognosis of severe primary infantile malnutrition. Br J Nutr.

[pmed-0040045-b018] Garrow JS, Smith R, Ward EE (1968). Electrolyte metabolism in severe infantile malnutrition.

[pmed-0040045-b019] Gómez F, Ramos-Galvan R, Frenk S, Cravioto JM, Chávez R (1956). Mortality in second and third degree malnutrition. J Trop Pediatr.

[pmed-0040045-b020] Waterlow JC (1992). Protein-energy malnutrition.

[pmed-0040045-b021] Golden MHN, Blaxter K, Waterlow JC (1985). The consequences of protein deficiency in man and its relationship to the features of kwashiorkor.

[pmed-0040045-b022] Heikens GT, Schofield WN, Dawson SM, Waterlow JC (1994). Long-stay versus short-stay hospital treatment of children suffering from severe protein-energy malnutrition. Eur J Clin Nutr.

[pmed-0040045-b023] Khanum S, Ashworth A, Huttly SRA (1994). Controlled trial of three approaches to the treatment for severe malnutrition. Lancet.

[pmed-0040045-b024] Collins S, Satler K (2002). The outpatient treatment of severe malnutrition during humanitarian relief programs. Lancet.

[pmed-0040045-b025] Schofield C, Ashworth A (1996). Why have mortality rates for severe malnutrition remained so high?. Bull World Health Organ.

[pmed-0040045-b026] World Health Organization (2003). Guidelines for the inpatient treatment of severely malnourished children. http://www.who.int/nutrition/publications/guide_inpatient_text.pdf.

[pmed-0040045-b027] Jackson AA, Ashworth A, Khanum S (2006). Improving child survival: Malnutrition Task Force and the paediatrician's responsibility. Arch Dis Child.

[pmed-0040045-b028] Kessler L, Daley H, Malenga G, Graham SM (2000). The impact of the human immunodeficiency virus type 1 on the management of severe malnutrition in Malawi. Ann Trop Paediatr.

[pmed-0040045-b029] Graham SM (2003). Impact of HIV on childhood respiratory illness: Differences between developing and developed countries. Pediatr Pulmonology.

[pmed-0040045-b030] Deen JL, Funk M, Guevara VC, Saloojee H, Doe JY Implementation of WHO guidelines on management of severe malnutrition in hospitals in Africa. Bull World Health Organ.

[pmed-0040045-b031] Brewster DR, Manary MJ, Graham SM (1997). Case management of kwashiorkor: An intervention project at 7 Nutritional Rehabilitation Centers in Malawi. Eur J Clin Nutr.

[pmed-0040045-b032] Morris JS, Molyneux EM (2003). Reduced mortality from severe protein-energy malnutrition following the introduction of WHO protocol in children in Malawi. Arch Dis Child.

[pmed-0040045-b033] Heikens GT (1995). Treatment of malnutrition. Lancet.

[pmed-0040045-b034] Sanders DM, Todd C, Chopra M (2005). Confronting Africa's health crisis: More of the same will not be enough. BMJ.

[pmed-0040045-b035] World Health Organization (2006). World Health Report 2006: Working together for health. http://www.who.int/whr/2006/en/.

[pmed-0040045-b036] Maitland K, Berkley JA, Shebbe M, Peshu N, English M (2006). Children with severe malnutrition: Can those at highest risk of death be identified with the WHO protocol?. PLoS Med.

[pmed-0040045-b037] English M, Esamai F, Wasunna A, Were F, Ogutu B (2004). Delivery of paediatric care at the first-referral level in Kenya. Lancet.

[pmed-0040045-b038] ALS Group (2005). Advanced paediatric life support: The practical approach.

[pmed-0040045-b039] Christie CDC, Heikens GT, Golden MHN (1992). Coagulase negative staphylococcal bacteraemia in severely malnourished Jamaican children. Ped Inf Dis J.

[pmed-0040045-b040] World Health Organization (2005). Pocketbook of hospital care for children: Guidelines for the management of common illnesses with limited resources.

[pmed-0040045-b041] Golden MHN (1988). The effects of malnutrition in the metabolism of children. Trans R Soc Trop Med Hyg.

[pmed-0040045-b042] Manary MJ, Brewster DR (2000). Intensive nursing care of kwashiorkor in Malawi. Acta Paediatr.

[pmed-0040045-b043] Waterlow JC (1986). Metabolic adaptation to low intakes of energy and protein. Ann Rev Nutr.

[pmed-0040045-b044] Jackson AA, Harrison GA, Waterlow JC (1990). The aetiology of kwashiorkor.

[pmed-0040045-b045] Nolan T, Angos P, Cunha AJ, Muhe L, Qazi S (2001). Quality of hospital care for seriously ill children in less-developed countries. Lancet.

[pmed-0040045-b046] Robertson MA, Molyneux EM (2001). Description of cause of serious illness and outcome in patients identified using ETAT guidelines in urban Malawi. Arch Dis Child.

[pmed-0040045-b047] Robertson MA, Molyneux EM (2001). Triage in the developing world—Can it be done?. Arch Dis Child.

[pmed-0040045-b048] Molyneux E, Ahmad S, Robertson A (2006). Improved triage and emergency care for children reduces inpatient mortality in resource constrained settings. Bull World Health Organ.

[pmed-0040045-b049] Molyneux EM, Webber MW (2004). Applying the right standards to improve hospital performance in Africa. Lancet.

[pmed-0040045-b050] Briend A, Prudhon C, Prinzo ZW, Dealmans B, Mason JB (2005). Putting back the management of severe malnutrition on the International Health Agenda. http://www.who.int/child-adolescent-health/New_Publications/NUTRITION/CBSM/Editorial.pdf.

[pmed-0040045-b051] The Bellagio Study Group on Child Survival (2003). Knowledge into action for child survival. Child Survival Series V. Lancet.

[pmed-0040045-b052] Duke T, Campbell H, Ayieko P, Opiyo N, English M (2006). Accessing and understanding the evidence. Bull World Health Organ.

[pmed-0040045-b053] Black RE, Morris SS, Bryce J (2003). Where and why are 10 million children dying every year? Child Survival Series I. Lancet.

